# The impacts of bronze age in the gene pool of Chinese: Insights from phylogeographics of Y-chromosomal haplogroup N1a2a-F1101

**DOI:** 10.3389/fgene.2023.1139722

**Published:** 2023-03-10

**Authors:** Hui-Xin Yu, Cheliger Ao, Xiao-Peng Wang, Xian-Peng Zhang, Jin Sun, Hui Li, Kai-Jun Liu, Lan-Hai Wei

**Affiliations:** ^1^ School of Ethnology and Anthropology, Institute of Humanities and Human Sciences, Inner Mongolia Normal University, Hohhot, China; ^2^ School of Management, Dalian University of Technology, Dalian, China; ^3^ School of Literature and Media, Xingyi Normal University for Nationalities, Xingyi, China; ^4^ MOE Key Laboratory of Contemporary Anthropology, School of Life Sciences, Fudan University, Shanghai, China; ^5^ B&R International Joint Laboratory for Eurasian Anthropology, Fudan University, Shanghai, China; ^6^ School of International Tourism and culture, Guizhou Normal University, Guiyang, China

**Keywords:** bronze age, Y-chromosome, N1a2a-F1101, Chinese, phylogeny

## Abstract

**Objectives:** Previous studies of archaeology and history suggested that the rise and prosperity of Bronze Age culture in East Asia had made essential contribution to the formation of early state and civilization in this region. However, the impacts in perspective of genetics remain ambiguous. Previous genetic researches indicated the Y-chromosome Q1a1a-M120 and N1a2a-F1101 may be the two most important paternal lineages among the Bronze Age people in ancient northwest China. Here, we investigated the 9,000-years history of haplogroup N1a2a-F1101 with revised phylogenetic tree and spatial autocorrelation analysis.

**Materials and Methods:** In this study, 229 sequences of N1a2a-F1101 were analyzed. We developed a highly-revised phylogenetic tree with age estimates for N1a2a-F1101. In addition, we also explored the geographical distribution of sub-lineages of N1a2a-F1101, and spatial autocorrelation analysis was conducted for each sub-branch.

**Results:** The initial differentiation location of N1a2a-F1101 and its most closely related branch, N1a2b-P43, a major lineage of Uralic-speaking populations in northern Eurasia, is likely the west part of northeast China. After ~4 thousand years of bottleneck effect period, haplgroup N1a2a-F1101 experienced continuous expansion during the Chalcolithic age (~ 4.5 kya to 4 kya) and Bronze age (~ 4 kya to 2.5 kya) in northern China. Ancient DNA evidence supported that this haplogroup is the lineage of ruling family of Zhou Dynasty (~ 3 kya-2.2 kya) of ancient China.

**Discussion:** In general, we proposed that the Bronze Age people in the border area between the eastern Eurasian steppe and northern China not only played a key role in promoting the early state and civilization of China, but also left significant traces in the gene pool of Chinese people.

## Introduction

Bronze Age globalization led to large-scale archaeological and cultural changes in much of Eurasia (de Barros Damgaard et al., 2018; Jeong et al., 2018; Jeong et al., 2019). The widespread spread of Indo-Europeans and related populations has been accompanied by strong replacement and mixing of population in many regions (Allentoft et al., 2015), such as South Siberia (Yu et al., 2020), Central Asia (Ning et al., 2019; Zhang et al., 2021; Kumar et al., 2022), South Asia (Narasimhan et al., 2019), the Middle East (Lazaridis et al., 2016), and Europe (Haak et al., 2015; Damgaard et al., 2018; Mathieson et al., 2018; Olalde et al., 2018). However, the Bronze Age globalization does not necessarily lead to the large-scale replacement of the population. We proposed that the subsistence strategy may be the key element. In northern Eurasia, Middle East, and East Asia, the natural environment may be not conducive to the original subsistence strategy, a. ka. The typical nomadic lifestyle, of Indo-Europeans. If the original subsistence strategy of Indo-Europeans can be firmly established in a certain region after the initial diffusion, significant population replacement may occur in this region and the local population may revive again.

In the central part of East Asia, during the Chalcolithic Age and the Bronze Age, early states and early civilizations arose (Liu and Chen, 2017). However, studies of bioarchaeology and ancient DNA have so far not observed obvious Indo-European-related genetic components in the central part of East Asia (Zhao et al., 2014; Ning et al., 2020; Yang et al., 2020; Mao et al., 2021; Robbeets et al., 2021; Wang et al., 2021; Xue et al., 2022). The demographic context of the relevant historical processes is unclear.

Previous studies have provided clues to population exchanges between the eastern Eurasian steppe region and the central part of East Asia. The Bronze Age populations of the Mongolian Plateau (∼4000–2500 YBP) is mainly a mixture of northern East Asian, Ancient North Eurasians, Southern Siberian indigenous and Indo-European related populations (Jeong et al., 2018; Jeong et al., 2020; Robbeets et al., 2021; Wang et al., 2021). The genetic contribution of these populations to the population of ancient central China was limited. Ancient DNA studies show that the genetic component of the Bronze Age populations in northwest China are basically local types, and there are no obvious foreign branches, like what had observed in Shimao (Xue et al., 2022), Jinchankou (Ning et al., 2020), and Xiajiadian sites (Li et al., 2011). On the other hand, archaeological studies confirm that the Mongolian Plateau is the intermediary zone where the bronze culture of central Eurasia spread to the central part of East Asia (Новгородова, 1989; Liu, 2004; Cai et al., 2007; Houle and Board., 2014; Liu and Chen, 2017). The important question raised by this is, is the spread of bronze culture to the Central Plains purely cultural transmission? What ancient people’s activities led to the emergence and prosperity of the bronze culture in northwest China? The relevant ancient DNA evidence is limit up till now.

Studies on the paternal Y chromosome provide some clues to the above questions. There is a high diversity of paternal components in the Chinese populations, and dozens of major haplogroups have been found (Zhong et al., 2010; Yan et al., 2014; Lu et al., 2020). Most of them originated from the central region of East Asia (Zhong et al., 2011). However, the demographic history of two of them, Q1a1a-M120 and N1a2a-F1101, shows links to Siberia and the eastern Eurasian steppe (Jeong et al., 2018; Sun et al., 2019; Jeong et al., 2020). Haplogroup Q-M242 has dozens of downstream clades, and only one Q1a1a-M120 clade appears frequently in East Asian populations (Huang et al., 2018; Wei et al., 2018; Sun et al., 2019). Ancient DNA studies suggest that haplogroup Q1a1a-M120 is the main paternal lineage of Bronze Age populations in northwest China (Zhao et al., 2014; Sun et al., 2019) and this lineage also appeared on the Mongolia Plateau (Jeong et al., 2018; Jeong et al., 2020). Previously, we had published a research to explore the migration from southern Siberia to East Asia and the role Q1a1a-M120 in the rise of the Bronze Culture populations in northwest China (Sun et al., 2019).

Previously, ancient DNA studies showed that N1a2a-F1101 was likely the paternal lineage of the royal family of the third dynasty of ancient China, the Zhou Dynasty (1027–256 BC) (Ma et al., 2021; Wei et al., 2022). The Zhou dynasty was the last dynasty of the Bronze Age in central China. Interestingly, N1a2b-P43, the mostly close branch of N1a2a-F1101, is the founder paternal lineage of the Uralic populations in northern Eurasia (Rootsi et al., 2007; Karmin et al., 2015; Ilumäe et al., 2016). The initial divergence of these two clades should have been between modern northern China and Siberia. Haplogroup N1a2a-F1101 is one of the major paternal lineage of modern Chinese, especially the Han Chinese (Liu et al., 2022; Tao et al., 2023). However, it remains unclear that how haplogroup N1a2a-F1101, a lineage likely originated in the north boundary region of China, become an important paternal component among ancient and modern populations in central China.

In this study, we analyzed the sequences and distribution of haplogroup N1a2a-F1101 within China. Our first objective was to construct a detailed phylogenetic tree for this paternal lineage with age estimation. Second, we analyzed the demographic history of this lineage and its role during the formation of early state and ancient civilization in ancient China. Specifically, we discussed the special demographic history in East Asia during the Bronze age globalization.

## Materials and methods

### Samples and sequencing

Saliva samples were collected from unrelated healthy males in East Asian populations over the past few decades. All participants provided written informed consent prior to participating. The study and sample collection process were reviewed and approved by the Medical Ethics Committee of Fudan University and Inner Mongolian Normal University, and complied with the ethical principles of the 2013 Helsinki Declaration of the World Medical Association. Genomic DNA was extracted using the DP-318 Kit (Tiangen Biotechnology, Beijing, China) according to the manufacturer’s protocol. A series of SNaPshot panels were used to determine the downstream Y-SNP (Single Nucleotide Polymorphism) haplgroup (Cai et al., 2011; Hu et al., 2015). As indicated by previous study, the major paternal Y-chromosome haplogroup of East Asia populations (like C, D, N, O. Q, and R) and downstream sub-branches can be identified by these panels (Cai et al., 2011; Hu et al., 2015). After that, we can select samples belonging to a certain haplogroup for further research. Extracted DNA of 229 individuals of haplogroup N1a2a-F1101 were sent for next-generation sequencing of the Y-chromosome using the Illumina HiSeq2000 platform (San Diego, CA, USA). All of these 229 individuals came from Chinese populations and most of them are Han Chinese (Supplementary Table S1). All of samples included in this study are healthy unrelated male individuals (Supplementary Table S2).

### Data availability statement

Following the regulations of the Human Genetic Resources Administration of China (HGRAC), the raw sequence data reported in this paper are available on request from the corresponding author. A list of variants of Y-SNP analyzed in this study was included in Supplementary Table S2.

### Data analysis

In total, 229 sequences of N1a2a-F1101 were analyzed (Supplementary Table S1). Read mapping and SNP (single nucleotide polymorphism) calling from next-generation sequencing data were conducted using standard procedures (BWA and SAMtools) and the human reference genome sequence hg38 (Li and Durbin, 2009; 2010). Bayesian evolutionary analyses were conducted using BEAST (v.2.0.0) (Bouckaert et al., 2014). To calculate divergence times in the phylogenetic tree, a point mutation rate of 0.74 × 10-9 per site per year (Karmin et al., 2015), inferred from the ∼12,000-year-old Anzick-1 male infant genome (Rasmussen et al., 2014), was applied. We referred to the regulations established by the Y Chromosome Consortium for the assignment of SNP and revised haplogroup names (The Y Chromosome Consortium, 2002) (The Y Chromosome Consortium, 2002). New haplogroup names for sub-branches of N1a2a-F1101 are listed in Supplementary Table S2.

To analyze the distribution of N1a2a-F1101 sub-lineages, Chinese participants were drawn from our inhouse database, which contains various types of information for each sample, including the Y haplogroup, native province, and native city. All steps followed those described in our previous study (Sun et al., 2021). In total, 1,601 Q1a1a-M120 individuals were identified among 140,900 Chinese males (total frequency: 2.54%, Supplementary Table S3). Frequency data were used to generate distribution maps using ArcGIS (version 10.3; Environmental Systems Research Institute Inc. Redlands, CA, USA). Please refer to the Supplementary Text of our previous study (Sun et al., 2021) for details about the ArcGIS was also used for a spatial autocorrelation analysis. The HotSpots plot indicate the clustering region of hop spots and cold spots which are generally corresponding to the center of diffusion and its mirror image region shown by the general distribution pattern. The Moran’s I Index indicate the degree of correlation between distribution frequency and spatial geographical distribution. The Lisa Cluster show the cluster and outlier of general distribution pattern of high-low frequency.

## Results

**FIGURE 1:**
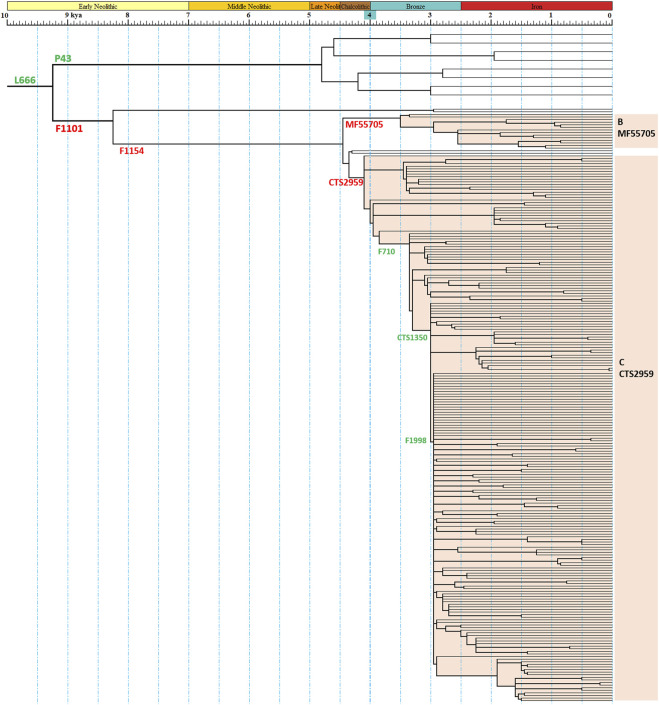
Schematic diagram of the revised phylogeny of haplogroup N1a2a-F1101. The red SNP labels indicated the definition marker of categories of sub-branches in this study. The green SNP labels indicated other important marker of the phylogenetic tree.

A revised phylogenetic tree of haplogroup N1a2a-F1101 were constructed with age estimation (Figure 1 and Supplementary Table S1). The haplogroups N1a2b-P43 and N1a2a-F1101 split at about 9300 years ago. There are similarities in the early history of the two haplogroups. They all experienced a very significant expansion after a bottleneck period of nearly 5,000 years and became the dominant paternal lineage of descendant populations. The main downstream branch of N1a2a-F1101 is N1a2a1-F1154, and the main differentiation node time is 4400 and 4000 years ago, and dozens of downstream branches are born. Among them, N1a2a1a1a1a1-F710 has undergone significant expansion after 3,350 years ago, giving birth to more than 70 downstream clades (Figure 1 and Supplementary Table S1). This topology suggests that the population expansion experienced by this paternal line around 3,000 years ago was the most significant of all paternal lineages in ancient East Asian populations at the same history period. Previously, ancient DNA studies suggested that this paternal line may be the paternal lineage of the Zhou Dynasty, the third dynasty of ancient China (Ma et al., 2021; Wei et al., 2022). The differentiation topology of this study supports the results of ancient DNA findings.

**FIGURE 2:**
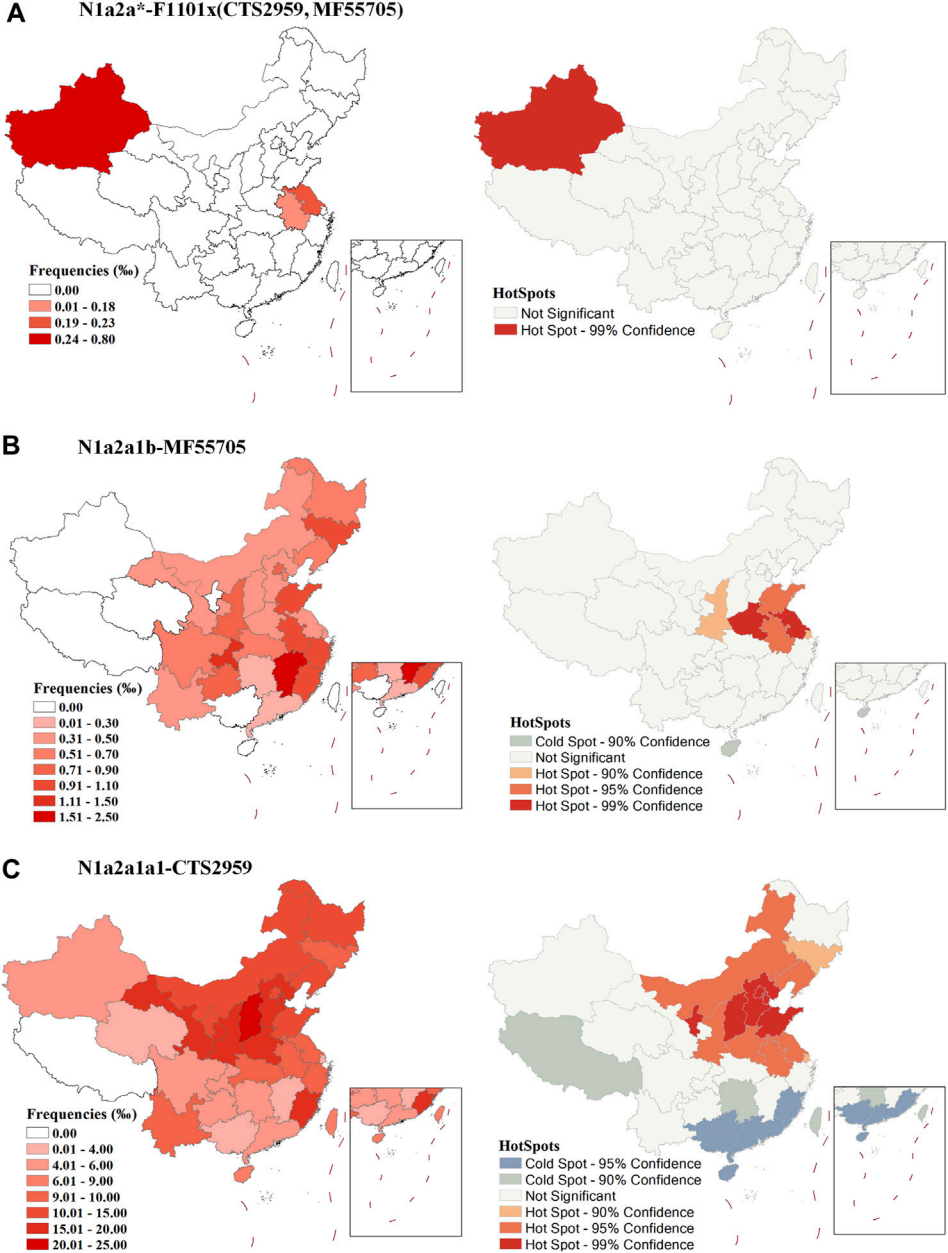
Geographic distribution of sub-branches of N1a2a-F1101 and results of a spatial autocorrelation analysis of frequencies. **(A)**: N1a2a*-F1101×(CTS2959,MF55705), **(B)**: N1a2a1b-MF55705, **(C)**: N1a2a1a1-CTS2959.

The proportion of early sub-branch N1a2a-F1101 clades in modern populations is very low and the distribution is discrete (Figure 2A, and Supplementary Table S2). Therefore, the possible diffusion centers shown by the HotSpots analysis in Figure 2A need to be treated with caution. Because sub-branch N1a2a1b-MF55705 is an early major branch of N1a2a-F1101 that emerged during the Chalcolithic Age (∼4400), we analyzed it separately. N1a2a1b-MF55705 is mainly distributed in eastern China (Figure 2B, and Supplementary Table S2). Results of spatial autocorrelation analysis suggests that the early spread center of this branch may have been the lower Yellow River region. Sub-branch N1a2a1a1-CTS2959 is the major downstream branch of N1a2a-F1101. High frequency of this lineage tend to found in northern China (Figure 2C, and Supplementary Table S2). HotSpots analysis in spatial autocorrelation analysis suggests that the early diffusion center of this clade may have been the central part of north China, consistent with the theory that this paternal line is the paternal lineage of the Zhou royal family.

## Discussion

### Early history between 9.3 kya and 4.4 kya

**FIGURE 3:**
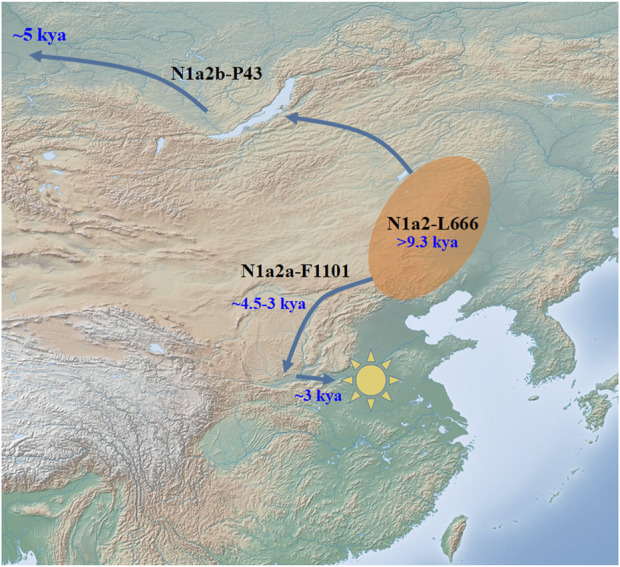
Possible diffusion pattern of N1a2a-F1101 in the past 9,000 years.

As the only two downstream clades of N1a2-L666, the geographical distribution of N1a2a-F1101 and N1a2b-P43 is very different from each other. Ancient DNA studies have identified early branches of N1a2a-F1101 and N1a2b-P43 in sites in the Baikal region (de Barros Damgaard et al., 2018; Kilinc et al., 2021; Ma et al., 2021). The most recent branch of N1a2-L666 is N1a1-M46, the main paternal type of the Uralic population (Ilumäe et al., 2016). The first two early branches under N1a1-M46, N1a1b-Y149447 and N1a1a3-F4065, are mainly distributed in northeast China (https://www.yfull.com/tree/N/) (Hu et al., 2015). Therefore, we speculate that the initial spread of haplogroup N1a2-L666 may have been in the southwestern part of northeastern China (Figure 3). We proposed that this region is also the initial diffusion center of N1a1-M46, while the diffusion of N1a1-M46 (>12 kya) happened earlier than that of N1a2-L666 (<9.3 kya) (Hu et al., 2015). In the early Holocene (about 11.2kya-8kya), with climate change and the rise of early agricultural populations in northern China, a part of the descendants of the ancestor group, representing by sub-lineage N1a2b-P43, spread to the high latitude region of Siberia, eventually becoming part of the Ural-speaking populations. The other part, representing by sub-lineage N1a2a-F1101, remained in the local area and participated in the formation of the northern Chinese populations in the later historical period (Figure 3).

A bottleneck period of 5,000 years was observed early in the evolution of N1a2a-F1101 (Figure 1, Supplementary Table S1). Similar lengthy bottleneck periods were observed in downstream structures of N1a2b-P43, N1a1-M46, and Q1a1a-M120 (Ilumäe et al., 2016; Sun et al., 2019). This evolutionary pattern is very different from the expansion pattern of ancient agricultural populations in East Asia, which continued to expand since the beginning of Neolithic age (Yan et al., 2014). The differentiation of the downstream clades of Q-M242 and N-231 presents a similar structure, i.e., downstream clades with high frequency distribution both in East Asia and Siberia, respectively. Therefore, we speculate that in the bottleneck interval, ancient populations with Q1a1a-M120 and N1a2a-F1101 as the main paternal lineages are likely to exist in the form of prehistoric hunter-gatherer populations in the border between the eastern Eurasian steppe and the northern-northeastern China. The drought and harsh natural environment of this area had a great influence on the evolution of the two paternal lineages in later historical periods.

### Expansion during the chalcolithic age and bronze age

During the Chalcolithic age (about 4.5 kya-4.0 kya) in East Asia, copper, cattle and wheat were introduced to the East Asian heartland (Liu and Chen, 2003; Liu, 2004; Liu and Chen, 2017). Archaeologists have suggested that the elements may have spread from northern boundary of China through the Eurasian steppe. However, the demographic context of this important cultural process is very ambiguous. Around 4,000 years ago, the Bronze culture arose in the agro-pastoral region of northwestern China and later spread across East Asia and Southeast Asia. The mixing of the bronze culture of agriculture and animal husbandry with the people of the middle and lower reaches of the Yellow River contributed to the establishment of three dynasties of the Bronze Age in ancient China, namely the Xia, Shang and Zhou dynasties (Liu and Chen, 2003; Liu, 2004; Liu and Chen, 2017).

As discussed above, ancient populations with Q1a1a-M120 and N1a2a-F1101 as the main paternal lineages may have played a mediating role in the spread of the Copper and Bronze cultures from the eastern Eurasian steppe to the central East Asian region, due to their area of activity in the junction zone. Due to the same reason, these two paternal lines experienced a very significant spread during the Bronze Age, becoming important patrilineal lineages that occupied an upper political position in the Bronze Age, and were frequently detected in the tombs of chiefs and nobles of the time (Zhao et al., 2014; Sun et al., 2019; Ma et al., 2021; Wei et al., 2022). An interesting thing is that the significant expansion of N1a2a-F1101 occurred after 3,300 years ago, significantly later than the major expansion period of Q1a1a-M120 (4.2 kya-3 kya, Figure 1). Nevertheless, several downstream clades of Q1a1a-M120, like F4759 and F4689, exhibit simultaneous expansion with N1a2a1a1a1a1-F710 (Sun et al., 2019). Ancient DNA data suggest that these two paternal lineages were concentrated in ancient populations in northwest China, and co-occurred in some tombs (Zhao et al., 2014; Ma et al., 2021; Wei et al., 2022). These ancient DNA studies also suggest that N1a2a-F1101 is likely the paternal lineage of the royal family of the Zhou Dynasty, while Q1a1a-M120 is the main paternal lineage of the Rong-Di populations (Means “Barbarians” in ancient Chinese). Both paternal lineages became the main paternal component of the Chinese group in later generations. In conclusion, we speculate that Q1a1a-M120 and N1a2a-F1101 together constitute the main paternal lineages of the populations that worked as farmers and pastoralists in northwest China during the Copper-Bronze Age. They played a key role in the emergence of bronze culture, early states, and early civilizations in central region of ancient China.

### Bronze age globalization in East Asia

As, discussed in the *Introduction* section, Bronze age globalization has led to mass replacement and mixing of populations in multiple parts of Eurasia (Allentoft et al., 2015). In East Asia, however, the situation is quite different. Ancient DNA shows that during the Copper-Bronze Age, the populations in the central East Asian region did not experience large-scale replacement, and the genetic components from Indo-Europeans are nearly absent. Based on previous literature and the results of this paper, we suggest that the Gobi Desert on the border between China and Mongolia may have hindered the spread of the Bronze culture and Indo-European-related populations. The hunter-gatherer communities that originally operated in the north and south of the Gobi Desert relied on their familiarity with the environment and long-distance material exchange networks to spread relevant cultural elements as intermediaries. In later historical periods, they became the main founders of the bronze culture populations in northwest China. These demographic histories led to the spread of Bronze culture into central East Asia as a form of cultural diffusion, unlike what happened in other parts of Eurasia during the Bronze Age period of globalization.

In summary, we constructed a high-resolution phylogeny for Y-chromosome haplogroup N1a2a-F1101, one of main paternal lineages of modern Chinese. We explored the demographic of this paternal haplogroup in the past 9,000 years. We also discussed the activity of ancient populations with this lineage and their role during the appearance of Bronze Age culture, the formation of early state and early civilizations in central region of China. The newly-discovered sub-branches and variants will assist in exploring the formation process of gene pool of Chinese populations and their cultural traditions.

## Data Availability

Following the regulations of the Human Genetic Resources Administration of China (HGRAC), the raw sequence data reported in this paper are available on request from the corresponding author. A list of variants of Y-SNP analyzed in this study was included in Supplementary Table S2, which is sufficient for reader to repeat the analyses of this study.
