# Supplementary figures and images for: The impacts of bronze age in the gene pool of Chinese: Insights from phylogeographics of Y-chromosomal haplogroup N1a2a-F1101
Source: Front Genet. 2023 Mar 10;14:1139722. doi: 10.3389/fgene.2023.1139722 (PMC10036388; doi:10.3389/fgene.2023.1139722)

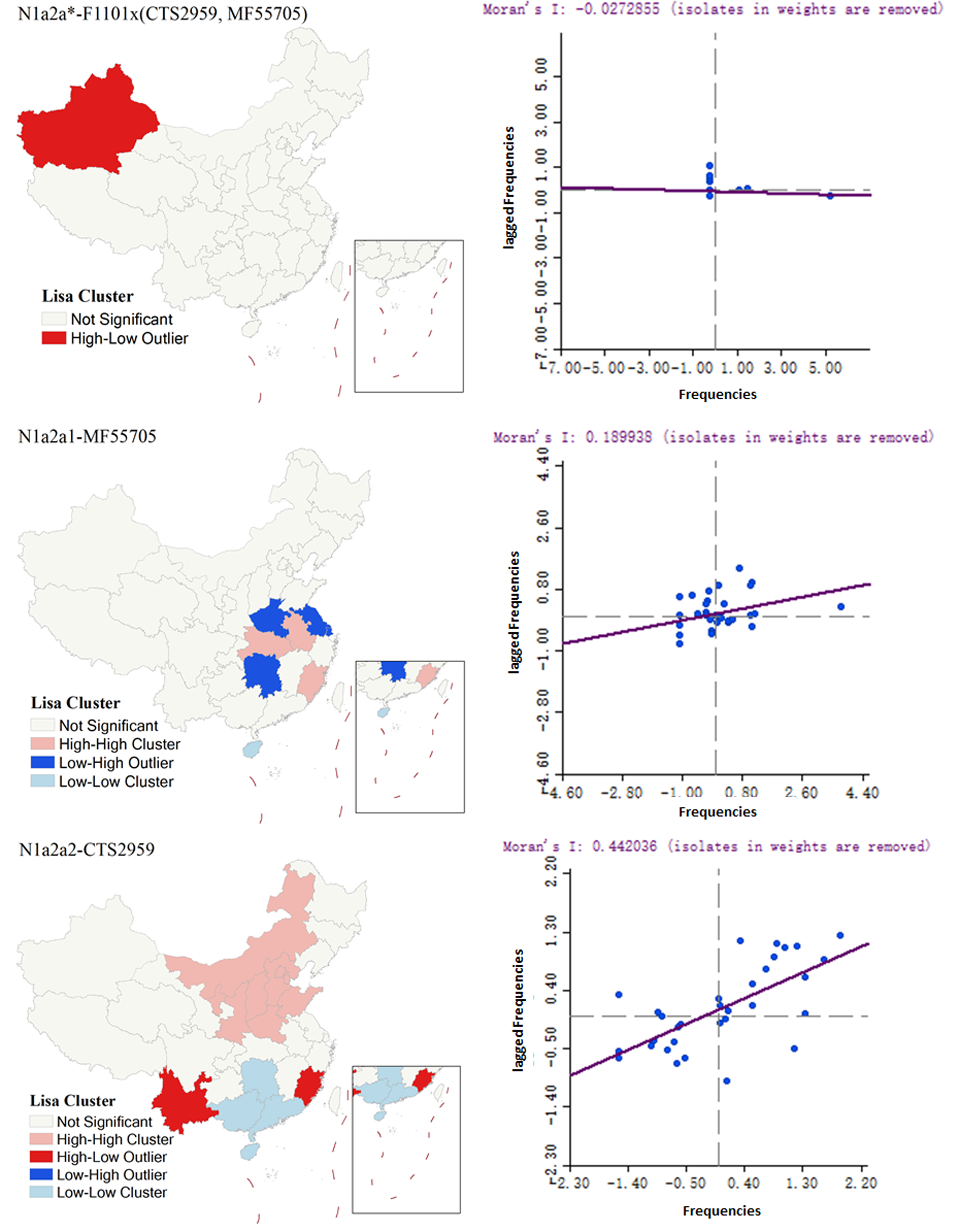

Supplement: Supplementary file 3 [file Image1.TIF]
